# Population-Level Density Dependence Influences the Origin and Maintenance of Parental Care

**DOI:** 10.1371/journal.pone.0153839

**Published:** 2016-04-19

**Authors:** Elijah Reyes, Patsy Thrasher, Michael B. Bonsall, Hope Klug

**Affiliations:** 1 Department of Biology, Geology, and Environmental Science, University of Tennessee at Chattanooga, Chattanooga, Tennessee, United States of America; 2 Mathematical Ecology Research Group, Department of Zoology, University of Oxford, Oxford, United Kingdom; 3 St Peter’s College, University of Oxford, Oxford, United Kingdom; University of Arkansas, UNITED STATES

## Abstract

Parental care is a defining feature of animal breeding systems. We now know that both basic life-history characteristics and ecological factors influence the evolution of care. However, relatively little is known about how these factors interact to influence the origin and maintenance of care. Here, we expand upon previous work and explore the relationship between basic life-history characteristics (stage-specific rates of mortality and maturation) and the fitness benefits associated with the origin and the maintenance of parental care for two broad ecological scenarios: the scenario in which egg survival is density dependent and the case in which adult survival is density dependent. Our findings suggest that high offspring need is likely critical in driving the origin, but not the maintenance, of parental care regardless of whether density dependence acts on egg or adult survival. In general, parental care is more likely to result in greater fitness benefits when baseline adult mortality is low if 1) egg survival is density dependent or 2) adult mortality is density dependent and mutant density is relatively high. When density dependence acts on egg mortality, low rates of egg maturation and high egg densities are less likely to lead to strong fitness benefits of care. However, when density dependence acts on adult mortality, high levels of egg maturation and increasing adult densities are less likely to maintain care. Juvenile survival has relatively little, if any, effect on the origin and maintenance of egg-only care. More generally, our results suggest that the evolution of parental care will be influenced by an organism’s entire life history characteristics, the stage at which density dependence acts, and whether care is originating or being maintained.

## Introduction

Parental care is a key life-history trait that influences sex roles, mating systems, and population dynamics [1–11]. Understanding the evolution of parental care has been a major focus in studies of behavioral ecology, and, more generally, recent work illustrates that the evolution of care is critical for understanding the evolution of social behavior [4, 8]. Parental care is expected to evolve when the fitness benefits of care outweigh the costs. Benefits of parental care in nature are diverse and can be associated with increased offspring size, increased offspring survival, increased offspring mating success, and/or altered offspring development [7, 12, 13]. Parental care is also associated with costs to parents, which can include a reduction in potential mating opportunities, survival, and fecundity [12, 14].

Whether parental care leads to net fitness benefits, though, will depend on an organism’s life-history characteristics (e.g. stage-specific mortality, maturation rates) and on the ecological conditions experienced (e.g. density dependent survival, competition for resources) [5, 7, 15–17]. Basic life-history characteristics are expected to influence the fitness benefits associated with parental care (e.g. [18]). For example, previous work suggests that parental care is most likely to originate and be beneficial when offspring need care the most (i.e. when offspring mortality in the absence of care is high [1, 3, 17, 19–21]. Likewise, offspring developmental rate can affect the evolution of parental care (reviewed in [13]). For example, if egg mortality is relatively high in the absence of care, parental care of eggs is likely to result in greater fitness benefits when the egg stage is relatively long in duration [22–24]. Additionally, other recent work suggests that parental care can be favored if care alters development, such that offspring spend less time in relatively dangerous life-history stages [13]. Finally, future potential reproductive opportunity is expected to affect investment in current offspring, and specifically, parents are expected to invest more in current offspring if they have relatively little expected future reproductive potential [25, 26]. For example, in some cases, parental care is more likely to originate when adult death rate is relatively high [17], as high adult mortality reduces future reproductive opportunities.

In addition to basic life-history characteristics, the ecological conditions that an organism experiences are also expected to influence the evolution of parental care [18]. For example, Wilson [27] and Clutton-Brock [1] hypothesized that parental care will be most likely to occur in organisms that experience relatively harsh environmental conditions and when resource competition is relatively high. Likewise, Thiel [28] hypothesized that the evolution of extended parental care is affected by ecological constraints, including predation and food availability. Empirically, ecology can have strong influences on parental care. For instance, a comparative analysis between two Peruvian poisonous frogs revealed a key ecological factor, breeding pool size, as the predominant driver influencing differences in the evolution of parental care [29].

We now have a sense of the basic life-history characteristics and, to some extent, the general ecological conditions that favor the evolution of parental care. However, we know relatively little about how ecology and basic life history interact to influence the evolution of parental care. In particular, population-level competition at various life-history stages (e.g. egg or adult stages) is likely to lead to density dependent survival or reproduction. Such density dependence is expected to influence the conditions under which care can evolve [30], and more specifically, we hypothesize that the life-history stage at which density dependence acts will affect the basic life-history conditions (stage specific rates of mortality and maturation) that promote the evolution of care.

Density dependence is known to act at various life-history stages in a range of organisms. For example, egg survival is known to be density dependent at the population level in copepods [31] and at the nest-specific level in fishes [32, 33] and beetles [34]. In the sand goby, *Pomastoschistus minutus*, for instance, density dependent egg survival occurs during the egg stage when males are caring for eggs in nests that are constructed under rocks or shells, and this density dependent egg survival is hypothesized to be related to the spread of disease [32]. In beaugregory damselfish, *Stegastes leucostictus*, density dependent egg mortality is related to oxygen availability, and low oxygen availability can lead to density dependent egg survival within nests in this species [33]. Likewise, juvenile survival is density dependent on a local scale in salmon, and this density dependence is associated with high metabolic or high predation costs associated with longer-distance dispersal that occurs when density is high [35]. In the red deer, *Cervus elphasus*, density dependent juvenile survival occurs at the population level [36], and in two species of damselfishes, *Dascyllus flavicaudus* and *D*. *trimaculatus*, density dependent juvenile mortality is related to competition for shelter among individuals in a given area [37]. With regard to the adult stage, density dependent mortality occurs in relation to food availability in Serengeti wildebeest (*Connochaetes taurinus*) [38] and at the population level in the marine goby *Coryphopterus glaucofraenum* [39]. Indeed, density dependent mortality has been well studied for decades in relation to population-level ecology [40], and as such, it is perhaps surprising that density dependent mortality has received relatively little attention in relation to the evolution of behavior such as parental care. As density dependent mortality affects the reproductive value of each individual, density dependent mortality is likely to influence the conditions under which parental care is most likely to result in fitness benefits in a given system.

Here, we use a theoretical approach to examine the effect of varying density dependent scenarios on the origin and maintenance of parental care. Specifically, we explore whether the basic life-history conditions (stage-specific rates of mortality and maturation) under which care is most likely to originate and be maintained (i.e. the life-history conditions under which care is expected to result in relatively high fitness relative to a no-care strategy) depends on whether density dependence acts on egg versus adult mortality. We focus on egg and adult mortality, as we consider this a feasible starting point to begin exploring the effects of stage-specific density dependence on the evolution of parental care. In doing so, we broadly explore the role that general ecology might play in influencing the evolution of parental care.

As mentioned above, we focus explicitly on both the origin and maintenance of parental care. Such a focus is critical, as the life-history conditions that favor the origin and maintenance of parental care (i.e. the life-history conditions that result in care being selected for) might differ (discussed in [7]). For example, in the early stages of the origin of parental care, we would expect individuals who exhibit care to be relatively rare in the population; as such, these individuals would primarily be competing with individuals who do not provide care to offspring. In contrast, once care has begun to spread in a population, the maintenance of care will be affected by competition that is occurring between individuals who exhibit care and those who do not. We hypothesize that such differences in competition associated with the origin versus the maintenance of care will affect the conditions under which care is most strongly selected for. By examining the link between density dependence and the basic life-history conditions under which care is under positive selection, and by explicitly considering both the origin and maintenance of care within a single theoretical framework, our work will provide general and testable predictions regarding when parental care is most likely to be selected for and originate and be maintained in various systems.

## Methods

### Model overview

Expanding upon our previous evolutionary ecology models of parental care (e.g. [5, 10, 11, 13, 16, 17, 41]), we model a population that consists of a resident strategy in which individuals do not exhibit parental care and an alternative rare (mutant) strategy in which individuals provide parental care. The alternative strategy, care, can be thought of as a phenotype that has arisen in a population due to mutation(s). We then explore the conditions under which care will be most likely to spread into the no-care strategy, and specifically, we identify the conditions under which care will result in a greater growth rate relative to the no-care strategy. Because the rate of increase of a strategy in a population provides insight into whether a strategy is spreading in the population, we use the growth rate of the mutant strategy as our measure of fitness (i.e. we identify the conditions under which care will be selected for and increase in the population relative to the no-care strategy; discussed further below). As is standard for invasion analyses [42], the resident strategy (no care) is assumed to be in equilibrium and the mutant strategy (care) is assumed to be rare in the population when we consider the origin of care. For the origin scenario, we therefore examine the fitness benefit associated with care (i.e. the growth rate of the care strategy relative to that of the non-care strategy) when the mutant is assumed to be very rare in the population. When we consider the maintenance of parental care, on the other hand, the mutant is assumed to have increased in density (i.e. parental care has begun to spread in the population and the density of rare mutants has increased relative to the origin scenario); as such, we consider the fitness benefit associated with care for varying levels of mutant densities (i.e. varying density levels of individuals that exhibit parental care). For both origin and maintenance scenarios, the density of the resident strategy is assumed to be at equilibrium in the population (i.e. the growth rate of the resident is assumed to be zero), and we assume a stage-structured life-history pattern, such that individuals pass through egg and juvenile stages and then mature and potentially reproduce as adults. Both the mutant and the resident strategies are assumed to experience the same baseline conditions—i.e. the same egg, juvenile, and adult mortalities and rates of maturation and reproduction before any costs and benefits of care are accounted for—in all cases. The mutant individuals, who provide care, then experience some costs and benefits of care, which are incorporated into the model through trade-off functions (Table 1; described below).

**Table 1:** Life history trade-offs associated with parental care (*c*) and initial investment in eggs (*1-d*_*E0*_) and (*1-d*_*Em0*_*)*.

Parameter	Case:	
	Resident Strategy: No care	Mutant Strategy: Parental care of eggs
Egg death rate (*d*_*E*_ and *d*_*Em*_)	*d*_*E*_ *= d*_*E0*_	Egg death rate ↓ as care ↑, such that *d*_*Em*_ *= d*_*Em0*_*·exp(-2·c)*
Reproductive rate (*r* and *r*_*m*_)	Adult reproductive rate ↓ as initial egg investment ↑, such that *r = r*_*0*_*·exp(-(1- d*_*E0*_*))*	Adult reproductive rate ↓ as initial egg investment ↑ and as care ↑, such that *r*_*m*_ *= r*_*m0*_*·exp(-((1- d*_*Em0*_*)+c))*
Adult death rate (*d*_*A*_ and *d*_*Am*_*)*	Adult death rate ↑ as initial egg investment ↑, such that *d*_*A*_ *= 1-[(1-d*_*A0*_*)·exp(-(1- d*_*E0*_*))]*	Adult death rate ↑ as initial egg investment ↑ and as care ↑ such that *d*_*Am*_ *= 1-[(1-d*_*Am0*_*)·exp(-((1- d*_*Em0*_*)+c))]*

As mentioned in the Introduction, we consider two population-level competition scenarios: 1) egg mortality is density dependent (i.e. average egg death rate depends on the density of eggs in the population) or 2) adult mortality is density dependent (i.e. average adult death rate depends on the absolute density of adults in the population). As the model makes no explicit assumptions about the spatial distribution of individuals in the population, density dependent mortality can be thought of as reflecting either 1) overall density dependent mortality that is occurring among all individuals of a given life-history stage in the population, which would apply if, for example, all adults within a population are competing for food or shelter or 2) an average density dependent mortality rate that individuals of a given life-history stage experience within a population, which, for example, would apply if the eggs in a population are clumped in different locations throughout the population at some consistent density and experience some overall level of density dependent mortality due to predation, disease spread, or oxygen availability. In other words, because our model lacks spatial structure, the density dependent mortality considered is reflective of either density dependence that results from competition among all members of a population or an average level of density dependence that occurs when subsets of the population (e.g. eggs in a nest) compete with one another for resources. Importantly, though, our model does not consider local density dependence that occurs when eggs are occurring at different densities throughout the population (i.e. our modeling approach does not account for variation in density dependence that will occur within a population).

For the origin of care scenario, the mutant is assumed to be very rare and the resident individuals are at equilibrium and are therefore the only source of competition for both the resident and the mutant individuals. In contrast, when we consider the maintenance of care, the mutant strategy is assumed to have increased in density in the population, and mutants and residents therefore experience competition with both mutant and resident individuals. For all scenarios considered (i.e. both the origin and maintenance of care, and the scenarios in which egg or adult mortality is density dependent) we quantify the fitness benefit of providing parental care (i.e. the fitness of care relative to the fitness of the no care strategy, which is measured as the growth or reproductive rate of the alternative care strategy relative to that of the no-care strategy; described below) with respect to basic life-history characteristics (i.e. egg, adult, and juvenile mortality and egg and juvenile maturation). In doing so, we identify the basic-life history characteristics (stage-specific mortality and maturation rates) under which parental care is most likely to result in positive fitness and therefore originate and be maintained for two broad ecological scenarios (egg and adult density dependent mortality). Below, we begin by outlining the equations that reflect mutant and resident strategy dynamics for the origin and maintenance of care scenarios for the cases in which adult or egg mortality is density dependent. We then describe how these equations can be used to calculate the growth rate associated with the care strategy relative to the growth rate of the no care strategy, which is our measure of fitness in this model (described further below).

### Resident Dynamics: Origin & Maintenance of Care

In all scenarios, individuals pass through egg (*E*) and juvenile stages, and then mature and potentially reproduce during the adult stage (*A*). When density dependence acts upon adult death rate, resident eggs increase as adults reproduce and decrease as eggs mature and as eggs die such that:

$$\frac{dE}{dt}=rA(t)-E(t)(d_{E}+m_{E})$$ (1)

where *r* is the reproductive rate (i.e. average egg fertilization rate in the population), *m*_*E*_ is the maturation rate of eggs (i.e. the rate at which individuals mature and pass from the egg to the juvenile stage), and *d*_*E*_ is the egg death rate (i.e. the rate at which individual eggs die).

Resident-strategy adults in the population increase as eggs mature and decrease as adults die, such that:

$$\frac{dA}{dt}=m_{E}E(t-\tau_{J})\sigma_{J}-d_{A}A^{2}(t)$$ (2)

where *σ*_*J*_ is the level of juvenile survival (i.e. the rate at which juvenile individuals survive), *τ*_*J*_ is the duration of the juvenile stage (i.e. the length of time spent as a juvenile), and *d*_*A*_ is adult death rate (i.e. the rate at which individuals in the population die as adults). In the scenario above, density dependence acting on adult mortality is incorporated through the term *d*_*A*_*A*^*2*^*(t)*, such that adult death rate increases non-linearly as adult density at a given time (*t*) increases (e.g. [43]). As we are considering the origin of care in this scenario, resident adults only experience competition with other resident individuals (i.e. adult death rate is unaffected by mutant density; Eq 2).

For the scenario in which parental care has originated in a population and is potentially being maintained evolutionarily, the mutant density is no longer negligible. As a result, competition now exists between the resident and the mutant individuals. As such, when the density dependence acts upon adult death rate, the resident adult death rate term is now –*d*_*A*_*A*(*t*)(*A*(*t*)+*A*_*m*_(*t*)), yielding the following equation:

$$\frac{dA}{dt}=m_{E}(E(t-\tau_{J})\sigma_{J})-d_{A}A(t)(A(t)+A_{m}(t)).$$ (3)

In this case, the resident’s death rate is now affected by competition with both the resident- and the mutant-strategy individuals. Mutant adult density, *A*_*m*_*(t)*, is a term that can now be varied, which allows us to examine how the fitness associated with care will change as mutant adult density changes (i.e. as parental care begins to spread and increase in frequency in the population). When we consider the maintenance of care, resident egg dynamics remain the same as in Eq 1.

The resident equilibria densities when density dependence is acting upon adult death rate are thus:

$$E^{*}=\frac{rA^{*}}{d_{E}+m_{E}}$$ (4)

for both the origin and maintenance scenarios and

$$A^{*}=\left(\frac{m_{E}r\sigma_{J}}{\left(d_{E}+m_{E}\right)}\right)\left(\frac{1}{d_{A}}\right)$$ (5)

for the origin of care scenario and

$$A^{*}=\frac{m_{E}\sigma_{J}r}{d_{A}(d_{E}+m_{E})}-A_{m}(t)$$ (6)

for the maintenance of care scenario.

When density dependence is acting upon egg death rate, resident eggs increase as resident adults reproduce and decrease as eggs mature and as eggs die such that:

$$\frac{dE}{dt}=rA(t)-m_{E}E(t)-d_{E}E{\left(t\right)}^{2}.$$ (7)

In this scenario, density dependent egg mortality is reflected through the term *d*_*E*_*E*^*2*^*(t)*, such that egg death rate increases non-linearly as egg density at a given time increases. Again, as we are considering the origin of parental care in this scenario, egg death rate is only affected by the density of resident (but not mutant) eggs, as mutants are assumed to be very rare during the early evolution of care (Eq 7). When we consider the maintenance of care for the density dependent egg death rate scenario, residents and mutants now experience competition with each other, such that the dynamics of the resident’s egg stage are given by:

$$\frac{dE}{dt}=rA(t)-m_{E}E(t)-d_{E}E(t)(E(t)+E_{m}(t))$$ (8)

where *E*_*m*_*(t)* is the density of mutant individuals in the population. Mutant density, *E*_*m*_*(t)*, is a term that can be varied, which allows us to examine the influence of a range of mutant densities on the fitness benefits associated with care once care has already originated in a population.

For the density dependent egg death rate scenario, adults in the population increase as eggs mature and decrease as adults die, such that:

$$\frac{dA}{dt}=m_{E}E(t-\tau_{J})\sigma_{J}-d_{A}A(t)$$ (9)

for both the origin and the maintenance of care scenarios.

The resident equilibria densities when density dependence is acting upon egg death rate are thus:

$$E^{*}=m_{E}\left(\frac{r\sigma_{J}}{d_{A}}-1\right)\left(\frac{1}{d_{E}}\right)$$ (10)

for the origin of care scenario,

$$E^{*}=\left(\frac{1}{d_{E}}\right)\left[m_{E}\left(\frac{r\sigma_{J}}{d_{A}}-1\right)\right]-E_{m}(t)$$ (11)

for the maintenance of care scenario, and

$$A^{*}=\frac{m_{E}E^{*}\sigma_{J}}{d_{A}}$$ (12)

for both the origin and the maintenance of care scenarios.

### Mutant Dynamics: Origin & Maintenance of Care

The dynamics of the mutant strategy, which is reflected by the subscript _*m*_ in the model, when density dependence is acting upon adult death rate are as follows for the origin of care scenario:

$$\frac{dE_{m}}{dt}=r_{m}A_{m}(t)-E_{m}(t)(d_{Em}+m_{Em})$$ (13)

$$\frac{dA_{m}}{dt}=m_{Em}E_{m}(t-\tau_{Jm})\sigma_{Jm}-d_{Am}A_{m}(t)(A^{*}+A_{m}(t))$$ (14)

where *r*_*m*_ is the mutant reproductive rate (i.e. average mutant egg fertilization rate in the population), *m*_*Em*_ is the mutant maturation rate of eggs (i.e. the rate at which mutant individuals mature and pass from the egg to the juvenile stage), *d*_*Em*_ is the mutant egg death rate (i.e. the rate at which mutant eggs die), *σ*_*Jm*_ is the rate of mutant juvenile survival (i.e. the rate at which juvenile mutants survive), *τ*_*Jm*_ is the duration of the mutant juvenile stage (i.e. the duration of time mutant individuals spend in the juvenile stage), and *d*_*Am*_ is mutant adult death rate (i.e. the rate at which mutant adults die). Under the origins of care scenario, the mutant is very rare and mutant density *A*_*m*_*(t)* is assumed to be negligible (*A*_*m*_*(t) = 0)*, and density dependent mutant adult death rate results only from competition with residents in the population, A*).

Using the mutant and resident dynamics, and by incorporating trade-offs reflecting costs and benefits of parental care (described below; Table 1), the invasion matrix for the origin of the alternative mutant strategy can be determined. The invasion matrix is a Jacobian matrix that incorporates the (linearized) dynamics of mutant strategies (described in the above equations); solving the characteristic equation of this matrix allows us to quantify the growth or reproductive rate of the mutant strategy relative to that of the resident strategy, and this allows us to determine the conditions under which care will increase in frequency and be favored in the population, i.e. the conditions under which the care strategy will have a positive growth rate (or positive fitness) relative to the resident strategy (see also general discussion of invasion matrices in Chapters 8 and 12 of [42] and additional discussion below).When density dependence acts on adult death rate, the invasion matrix associated with the origin of care is as follows:

$$\left(\begin{array}{cc} -d_{Em}-m_{Em}-\lambda & r_{m}\\ \sigma_{Jm}m_{Em}(1-\lambda \tau_{Jm}) & -d_{Am}A^{*}-\lambda \end{array}\right)$$ (15)

where A^*^ is the equilibrial adult density of the resident strategy and the dominant eigenvalue, *λ*, is the measure of the fitness, i.e. the growth or reproductive rate, of the mutant strategy relative to the resident strategy (see also Chapters 8 and 12 of [42] for additional details of the calculation of *λ*). Taking the determinant of this matrix (Eq 15) and solving the resulting characteristics equation:

$$(\lambda +d_{Em}+m_{Em})(\lambda +d_{Am}A^{*})+rm_{Em}(1-\lambda \tau_{Jm})\sigma_{Jm}=0$$ (16)

for the dominant eigenvalue *λ* then provides a measure of the fitness benefit that is associated with the origin of parental care for given parameters. Specifically, when *λ* is greater than zero, parental care has a greater growth (or reproductive) rate than the no-care strategy and can invade the population. In other words, a positive *λ* suggests that care will be increasing in frequency and favored in the population. While *λ* provides a measure of the growth rate of the mutant strategy in the population and as such, can be used as a measure of fitness, it is important to note that *λ* does not provide insight into the long-term adaptive dynamics of care (i.e. it does not provide information on whether care will persist or potentially co-exist over the long term with a no-care strategy).

When adult death rate is density-dependent and we consider the maintenance of care, mutants now also experience competition with both mutant and resident strategies, such that:

$$\frac{dA_{m}}{dt}=m_{Em}(E(t-\tau_{Jm})\sigma_{Jm})-d_{Am}A_{m}(t)(A^{*}+A_{m}(t)).$$ (17)

Given the above dynamics, the invasion matrix for the maintenance of care when adult death rate is density dependent is:

$$\left(\begin{array}{cc} -d_{Em}-m_{Em}-\lambda & r_{m}\\ \sigma_{Jm}m_{Em}(1-\lambda \tau_{Jm}) & -(d_{Am}A^{*}+2d_{Am}A_{m})-\lambda \end{array}\right).$$ (18)

Taking the determinant and solving the resulting characteristic equation:

$$(\lambda +d_{Em}+m_{Em})(\lambda +(d_{Am}A^{*}+2d_{Am}A_{m}))+r_{m}m_{Em}(1-\lambda \tau_{Jm})\sigma_{Jm}=0$$ (19)

for the dominant eigenvalue *λ* then provides a measure of the fitness benefit that is associated with the maintenance of parental care for given parameters across a range of mutant densities. In other words, *λ* provides a measure of fitness for care and indicates when care is most likely to increase in frequency in a population for varying levels of mutant densities. When *λ* is greater than zero, we would expect the strategy of parental care to be increasing in frequency and favored in the population.

When density dependence acts upon the egg death rate, the mutant dynamics for the origin of care are as follows:

$$\frac{dE_{m}}{dt}=r_{m}A_{m}(t)-m_{Em}E_{m}(t)-d_{Em}E_{m}(t)(E^{*}+E_{m}(t))$$ (20)

$$\frac{dA_{m}}{dt}=m_{Em}E_{m}(t-\tau_{Jm})\sigma_{Jm}-d_{Am}A_{m}(t)$$ (21)

where parameters are as defined above. Similar to density dependence acting on adult mortality, the mutant is very rare during the origin of care and mutant density *E*_*m*_*(t)* is assumed to be negligible (*E*_*m*_*(t) = 0)*, as density-dependent mutant egg death rate results only from competition with residents in the population, E*. For the case of density-dependent egg death rate, the invasion matrix for the origin of the alternative mutant strategy is thus:

$$\left(\begin{array}{cc} -d_{Em}E^{*}-m_{Em}-\lambda & r_{m}\\ \sigma_{Jm}m_{Em}(1-\lambda \tau_{Jm}) & -d_{Am}-\lambda \end{array}\right).$$ (22)

Again, solving the resulting characteristics equation:

$$(\lambda +d_{Em}E^{*}+m_{Em})(\lambda +d_{Am})+r_{m}m_{Em}(1-\lambda \tau_{Jm})\sigma_{Jm}=0$$ (23)

for the dominant eigenvalue *λ* then provides a measure of the fitness benefit that is associated with parental care for given parameters. When *λ* is greater than zero, we would expect parental care to increase in frequency in the population.

When we consider the density dependent egg death rate for the maintenance of care scenario, mutants now experience competition with mutant and resident individuals, such that:

$$\frac{dE_{m}}{dt}=r_{m}A_{m}(t)-m_{Em}E_{m}(t)-d_{Em}E_{m}(t)(E^{*}+E_{m}(t)).$$ (24)

Using these dynamics, the invasion matrix for the maintenance of parental care when density dependence is acting upon egg death rate is:

$$\left(\begin{array}{cc} -(d_{Em}E^{*}+2d_{Em}E_{m})-m_{Em}-\lambda & r_{m}\\ \sigma_{Jm}m_{Em}(1-\lambda \tau_{Jm}) & -d_{Am}-\lambda \end{array}\right).$$ (25)

Solving the resulting equation:

$$(\lambda +(d_{Em}E^{*}+2d_{Em}E_{m})+m_{Em})(\lambda +d_{Am})+r_{m}m_{Em}(1-\lambda \tau_{Jm})\sigma_{Jm}=0$$ (26)

for the dominant eigenvalue *λ* provides a measure of the fitness benefit that is associated with tparental care for given parameters across a range of mutant densities. When *λ* is greater than zero, care is increasing in frequency in the population and we would expect care to be maintained in the population.

For all parental care scenarios (i.e. the scenarios in which egg death rate or adult death rate is density dependent and for the origin and maintenance scenarios), we examined the fitness benefit (i.e. *λ*, described above) associated with parental care with respect to basic life-history characteristics (stage-specific mortality and maturation rates and egg and juvenile maturation) and changes in mutant density. In doing so, we identify the basic life-history conditions under which parental care will result in the greatest net fitness benefits as the mutant strategy spreads in the population (i.e. as egg and/or adult mutant density increases). Importantly, it is the qualitative patterns that we identify (i.e. the qualitative relationship between a given life-history trait and the fitness benefits of care) that are informative and provide insight into the general conditions that will allow care to spread in a population. The quantitative findings (e.g. the specific fitness value associated with a given analysis) are less informative, as the specific numeric fitness values will be sensitive to the parameter values considered. As such, when examining the results and figures presented below, it is the qualitative conditions (e.g. low or high life-history trait values) that result in the greatest fitness benefits of care that are most likely to allow the origin and/or maintenance of care.

### Costs and Benefits of Parental Care

Parents can affect offspring fitness by 1) investing energy and nutrients into eggs, which we refer to as initial egg allocation, and 2) providing post-oviposition parental care behavior to offspring in one or more life history stages, which we refer to simply as parental care [5, 17]. Both initial egg allocation and parental care be associated with benefits to offspring and costs to parents. Here, as in our previous work (e.g.[17]), we consider two general parental care strategies: no parental care (i.e. the resident strategy) and parental care of eggs (the mutant strategy). The different care strategies are represented in the model through the incorporation of trade-offs into the resident and mutant dynamics (described below and in Table 1). The level of parental care is approximated by a fixed value, *c* (Table 1), and this can be thought of as some average level of care that a mutant adult provides to its mutant offspring. Using this approach, care is assumed to be fixed and independent of offspring number. In other words, care can be thought of as being shared among offspring [1], and non-shareable, i.e. depreciable, care is something that we do not consider in the current model. Egg death rate is used as our proxy of initial egg allocation—i.e. if parents initially allocate a relatively large amount of resources into their eggs, those eggs are assumed to have relatively high survival. Parental care is assumed to increase offspring survival during the stage in which it is provided; in other words, as the level of care to eggs increases, egg survival increases (Table 1). Providing care behavior is assumed to be costly to parents, and as the level of care increases, adult survival declines (i.e. death rate increases) and reproductive rate decreases (Table 1). Likewise, initial egg allocation is also expected to be costly to parents, such that as initial egg allocation increases, adult survival and reproductive rates decrease (see also [17]).

In all cases, we assume non-linear trade-offs that are identical to trade-offs that we have used in our previous work (Table 1; see also [17]), as non-linear trade-offs capture a broader repertoire of plausible biological scenarios and have been used in previous models of parental investment and care [17, 23, 44]. Furthermore, focusing on these non-linear trade-offs allows us to compare the results of the current model to the results of previous work using a similar theoretical framework [17]. Importantly, these trade-off functions are expected to have large effects on the fitness of parental care, as these trade-offs influence reproduction and survival during different life-history stages. However, if differing trade-off functions are similar qualitatively, the patterns of when care will result in positive fitness will be qualitatively similar, regardless of whether the specific function is linear or non-linear [41]. If trade-off functions differ qualitatively, the results are likely to vary qualitatively. As such, it is important to recognize that the qualitative patterns associated with our models are likely to apply to systems in which 1) egg mortality decreases with increasing parental care and 2) adult survival and reproduction decrease with increasing investment in eggs and with increasing parental care. While some species might not meet the assumptions of our modeling trade-off functions, we view these as reasonable trade-off assumptions [1] that will allow for a first look at how density dependent mortality influences the fitness benefit associated with care.

## Results

**Fig 1:**
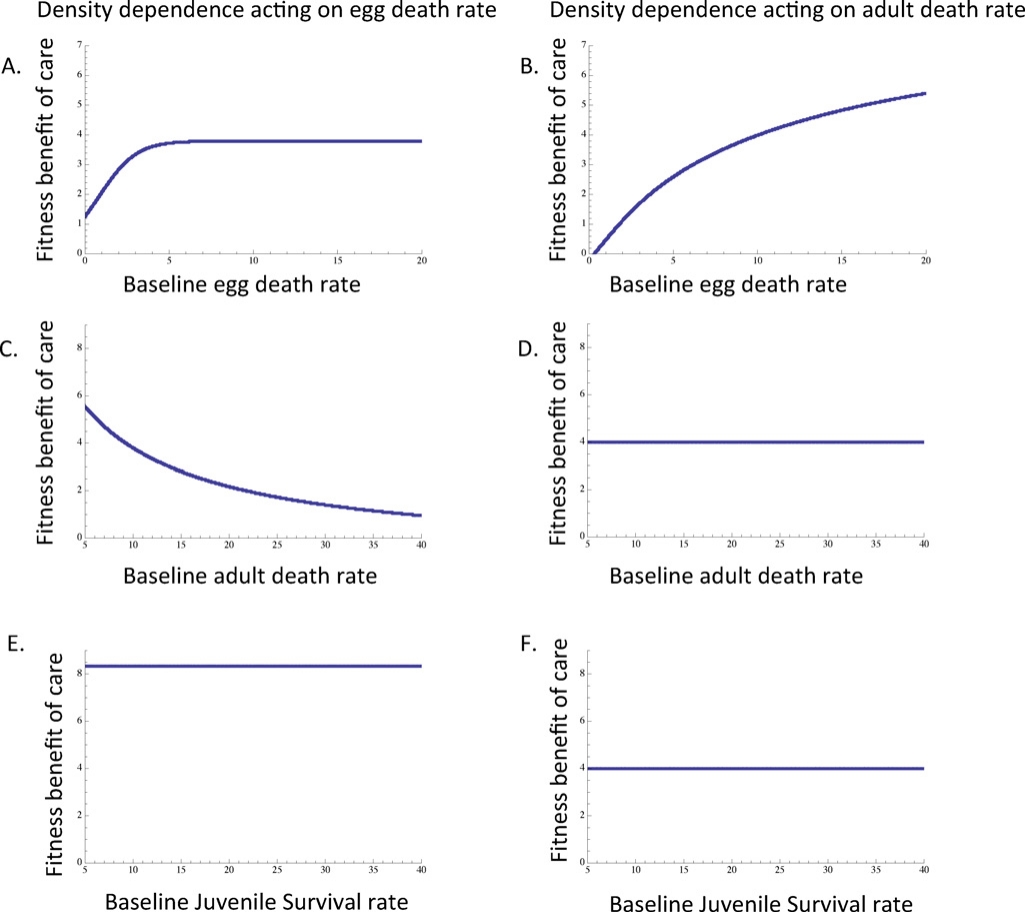
The origin of parental care in relation to egg, adult, and juvenile survival. When egg death rate is density-dependent (A) and when adult death rate is density dependent (B) the fitness benefit associated with care is greatest when baseline egg death rate (i.e. egg death rate before any care is accounted for) is relatively high. When egg death rate is density dependent (C), the fitness benefit associated with care is greatest when baseline adult death rate is relatively low. However, when the adult death rate is density dependent (D), there is no relationship between the fitness of care and baseline adult death rate. When egg death rate is density-dependent (E) and when adult death rate is density-dependent (F), the fitness benefit associated with care is unrelated to juvenile survival. Unless otherwise noted, *d*_*E* 0_ = *d*_*E m0*_ = 10, *m*_*E*_ = *m*_*E* m_ = 0.1, *r*_*0*_ = *r*_*m0*_ = 10,000, *d*_*A0*_ = *d*_*Am0*_ = 10, *σ*_*J0*_ = *σ*_*Jm0*_ = 0.01, *τ* = 0.1, c = 0.9.

**Fig 2:**
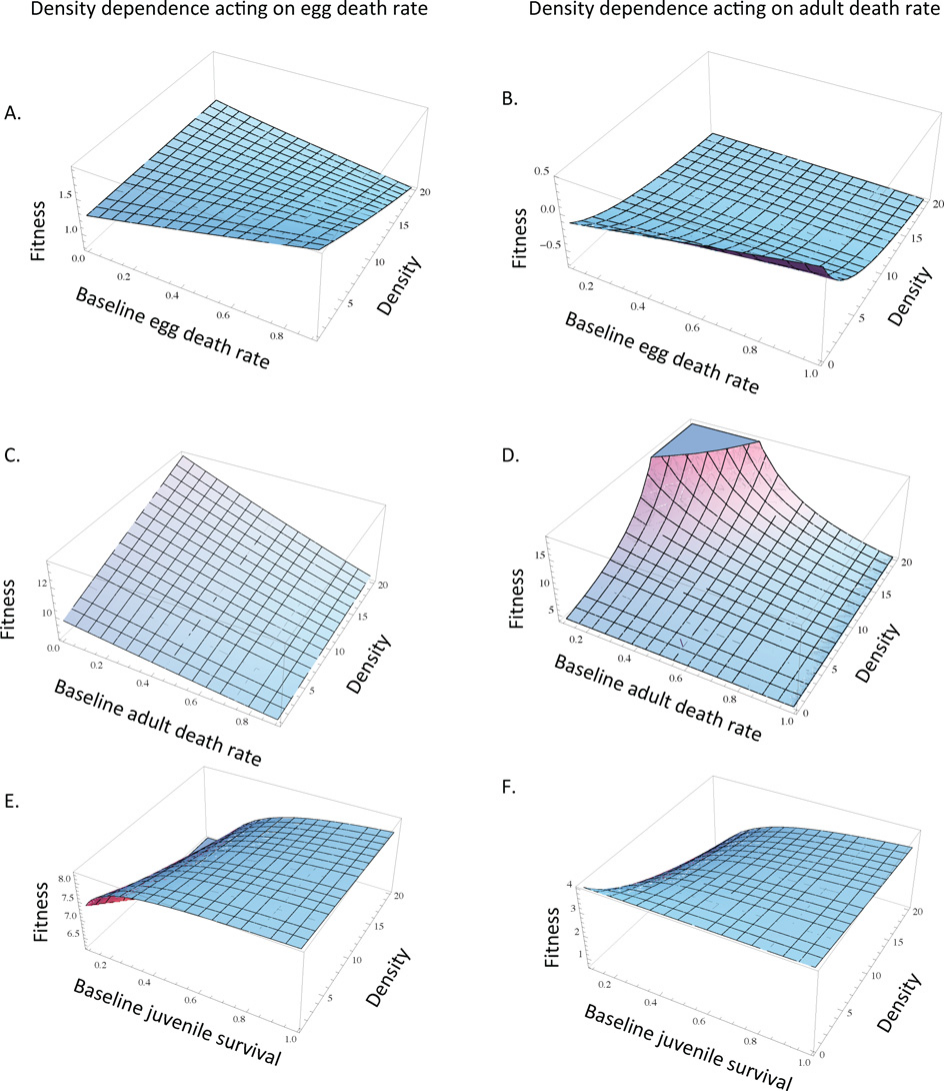
The maintenance of parental care in relation to egg, adult, and juvenile survival and the density of mutant individuals. When egg death rate (A) and when adult death rate (B) are density dependent, the fitness benefit associated with care is greatest at higher levels of baseline egg death rate when mutant density is relatively low. As density increases, the fitness benefits associated with care decrease and the fitness benefits of care become insensitive to baseline egg death rate (A-B). When density dependence acts on egg death rate, the fitness benefits associated with the maintenance of care are highest when adult death rate is relatively low (C). When density dependence acts on adult death rate, the fitness benefits associated with the maintenance of care are relatively insensitive to baseline adult death rate at low mutant densities (D). When density dependence acts on adult mortality and mutant density is high, the maintenance of parental care is most strongly favored at lower baseline adult death rates high (D). When mutant density is relatively high, the fitness benefits associated with the maintenance of care are greatest when juvenile survival is intermediate or relatively high regardless of whether density dependence acts on egg death rate (E) or adult mortality (F). Unless otherwise noted, *d*_*E* 0_ = *d*_*E m0*_ = 10, *m*_*E*_ = *m*_*E* m_ = 0.1, *r*_*0*_ = *r*_*m0*_ = 10,000, *d*_*A0*_ = *d*_*Am0*_ = 10, *σ*_*J0*_ = *σ*_*Jm0*_ = 0.01, *τ* = 0.1, c = 0.9.

**Fig 3:**
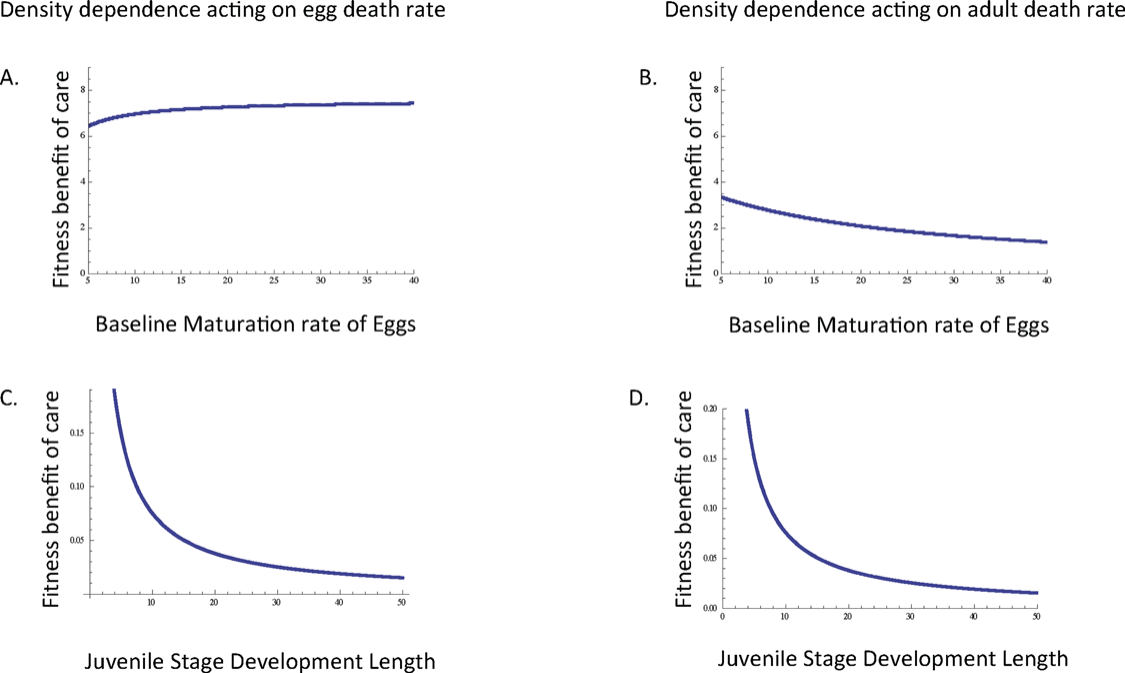
Fitness benefits of the origin of parental care in relation to egg and juvenile development. When egg death rate is density dependent (A), the fitness benefit associated with care is highest when egg maturation rate is relatively high, whereas when adult death rate is density dependent (B), the fitness benefit of care is highest when egg maturation rate is relatively low. However, in both cases (A-B) the slope of this relationship is close to zero, and as such, egg maturation rate has minimal effects on the fitness benefits of care. When egg death rate (C) and adult death rate (D) are density dependent, the fitness benefit associated with care is highest when the juvenile stage is relatively short. Unless otherwise noted, *d*_*E* 0_ = *d*_*E m0*_ = 10, *m*_*E*_ = *m*_*E* m_ = 0.1, *r*_*0*_ = *r*_*m0*_ = 10,000, *d*_*A0*_ = *d*_*Am0*_ = 10, *σ*_*J0*_ = *σ*_*Jm0*_ = 0.01, *τ* = 0.1, c = 0.9.

**Fig 4:**
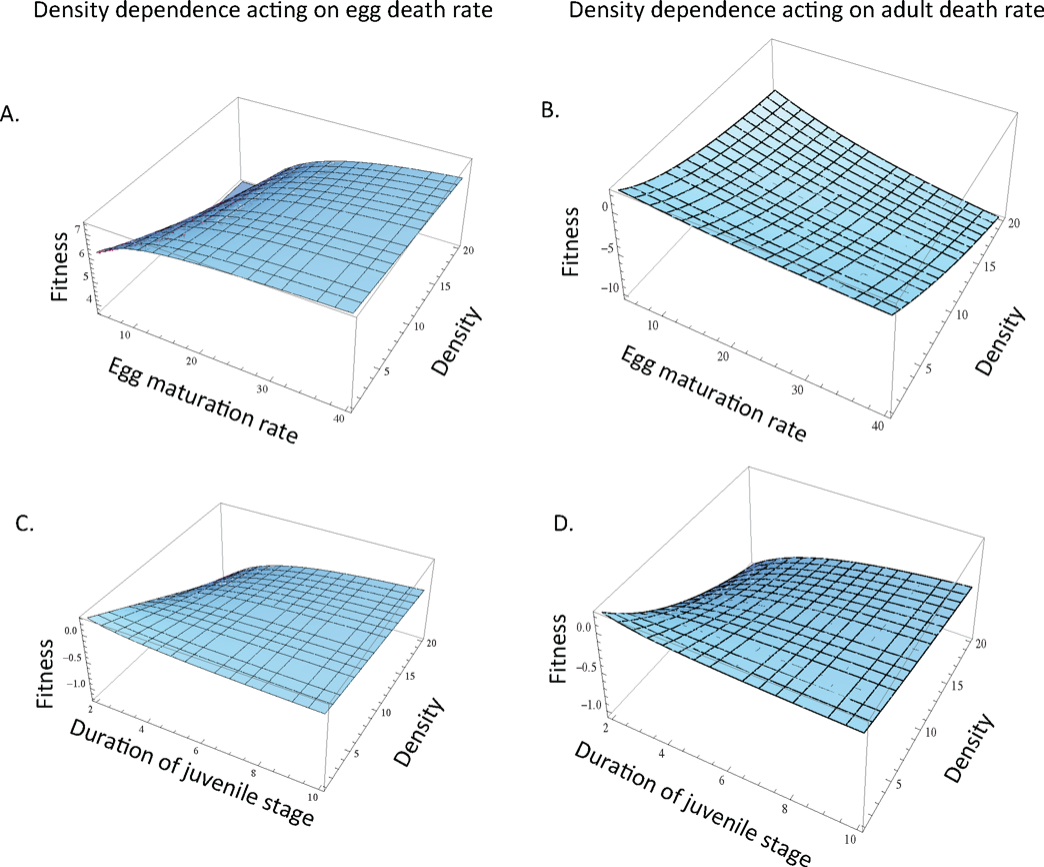
Fitness benefits of the maintenance of parental care in relation to egg and juvenile development and the population density of mutant individuals. When density dependence is acting upon the egg death rate, (A) the fitness benefit associated with the maintenance of care is highest when egg maturation rate is lowest and mutant density is low. When adult mortality is density dependent, the fitness benefits associated with the maintenance of care are greatest when egg maturation rate is low and mutant density is low. When egg death rate (C) and when adult death rate (D) are density dependent, the fitness benefits associated with the maintenance of care are greatest when the duration of the juvenile stage is relatively long if mutant density is relatively high. Unless otherwise noted, *d*_*E* 0_ = *d*_*E m0*_ = 10, *m*_*E*_ = *m*_*E* m_ = 0.1, *r*_*0*_ = *r*_*m0*_ = 10,000, *d*_*A0*_ = *d*_*Am0*_ = 10, *σ*_*J0*_ = *σ*_*Jm0*_ = 0.01, *τ* = 0.1, c = 0.9.

Whether density dependence acts on egg or adult death rate influences the life-history conditions under which parental care is expected to originate and be maintained in some, but not all, cases (Figs 1–4). Below, we outline the life-history conditions that favor the origin and maintenance of care, and we describe differences that arise when density-dependence acts on egg versus adult survival. We first focus on life-history traits associated with survival (i.e. egg, adult and juvenile survival) and then describe the patterns related to egg and juvenile maturation.

### Offspring need drives the origin, but not the maintenance, of parental care

The origin of parental care is most strongly favored when baseline egg death rate (i.e. egg death rate in the absence of any care) is relatively high, regardless of whether egg death rate (Fig 1A) or adult death rate (Fig 1B) is density-dependent. In the case of density-dependent egg death rate, the fitness benefit associated with the origin of care increases initially as baseline egg death rate increases, but then plateaus, suggesting that the fitness benefits of the origin of care are independent of baseline egg death rate when eggs have low survival in the absence of care (Fig 1A). In the case of density-dependent adult death rate, the fitness benefit associated with the origin of care continually increases with increasing baseline egg death rate (Fig 1B). In general, these patterns suggest that the origin of parental care is strongly favored when eggs have high mortality in the absence of parental care.

In contrast, when we focused on the maintenance, rather than the origin, of parental care, the fitness benefits associated with providing care are relatively insensitive to baseline egg death for the scenarios in which egg survival or adult survival is density dependent, particularly when mutant density is relatively high (Fig 2A and 2B). In general, under varying levels of baseline egg death rate, increases in the density of individuals exhibiting the mutant strategy (care) decrease the fitness benefits associated with the maintenance of care, particularly when density dependence acts on adult death rate (Fig 2A and 2B).

Together, these patterns suggest that high offspring need is critical in driving the origin of parental care, regardless of whether density dependence acts on adults or eggs (Fig 1A and 1B). In contrast, the maintenance of care is relatively insensitive to offspring need (Fig 2A and 2B). In other words, as parental care spreads in a population and the density of individuals exhibiting the mutant strategy increases, the fitness benefit associated with care is relatively insensitive to offspring need (Fig 2A and 2B).

### Density dependent mortality can affect the adult death rate scenarios under which care will be favored and maintained

When egg death rate is density dependent, the fitness benefits associated with the origin of care are highest when adult death rate is relatively low (Fig 1C). In contrast, when adult death rate is density dependent, there is no relationship between baseline adult death rate and the fitness benefits associated with the origin of care (Fig 1D).

Consistent with the origin of care scenario, the fitness benefits associated with the maintenance of care are greater when baseline adult death rate is relatively low for the scenario in which egg survival is density dependent (Fig 2C). This qualitative pattern remains consistent as the mutant strategy spreads in the population (i.e. care continues to be most strongly favored at lower baseline adult death rates as egg density increases in Fig 2C). In other words, parental care is likely to result in the greatest fitness benefits when adults have low mortality if egg survival is density dependent. When adult mortality is low, parents have higher potential for future reproduction (because they are less likely to die at a given point in time), and it is possible that the future reproductive potential of parents is influencing the link between adult mortality and the benefit of care in these scenarios.

Also consistent with the origin scenario, when we consider density dependence acting on adult mortality, the fitness benefits associated with the maintenance of parental care are insensitive to baseline adult death rate when mutant adult density is relatively low (Fig 2D). However, when density dependence acts on adult mortality and mutant density is high, the maintenance of parental care is most strongly favored at lower baseline adult death rates high (Fig 2D).

These results suggest that the role of baseline adult mortality in influencing the evolution of care will depend on the life-history stage that density dependence acts on, as well as the relative abundance of the mutant strategy in the population. In general, baseline adult mortality has greater influence on the fitness benefits associated with care when egg survival is density dependent (Fig 1C) and when adult mortality is density dependent and mutant density is relatively high (Fig 2D).

### Juvenile survival has little, if any, effect on the origin and maintenance of egg-only care

For both the scenario of density dependent egg death rate and of density dependent adult death rate, the fitness benefits associated with the origin of parental care are relatively insensitive to juvenile survival (Fig 1E and 1F). In general, this pattern is consistent when we consider the maintenance of care when mutant density is relatively low (Fig 2E and 2F); however, when mutant density is relatively high, the fitness benefits associated with the maintenance of care are greatest when juvenile survival is intermediate or relatively high regardless of whether density dependence acts on egg death rate or adult mortality (Fig 2E and 2F). Together, these patterns suggest that juvenile survival will have little effect on the origin of egg-only care regardless of the life-history stage that density dependence acts on, but that care is less likely to be maintained when mutant density is high and juvenile survival is low.

### Egg maturation rate has relatively little influence on the origin and maintenance of care

When density dependence acts on egg death rate, the fitness benefit associated with the origin of care is highest when egg maturation rate is large (Fig 3A), whereas when density dependence acts on adult death rate fitness is highest when egg maturation rate is lowest (Fig 3B). However, these effects are relatively small (Fig 3A and 3B), and as such, egg maturation rate has relatively little effect on the fitness associated with the origin of care.

The fitness benefits associated with the maintenance of care in relation to egg maturation rate depends on the type of density dependence and the density of mutants. When density dependence acts on egg mortality, low rates of egg maturation and high densities are less likely to maintain the fitness benefits of care (Fig 4A). In contrast, when density dependence acts on adult mortality, high levels of egg maturation and increasing densities are less likely to maintain care (Fig 4B).

### The effect of juvenile stage duration depends on the density of the mutant strategy

In the cases of both density dependent egg death rate and density dependent adult death rate, the fitness benefits associated with the origin of care are highest when the juvenile stage is relatively short (Fig 3C and 3D). With respect to the maintenance of parental care, the fitness benefits of care are relatively insensitive to juvenile stage duration when egg survival or adult mortality is density dependent if mutant density is low (Fig 4C and 4D). However, if mutant density is high, the maintenance of care is unlikely to be favored when the duration of the juvenile stage is relatively short (Fig 4C and 4D). Indeed, when mutant density is high, the fitness benefits associated with the maintenance of care are greatest when the duration of the juvenile stage is relatively long.

### Intermediate levels of care result in the greatest fitness benefit

**Fig 5:**
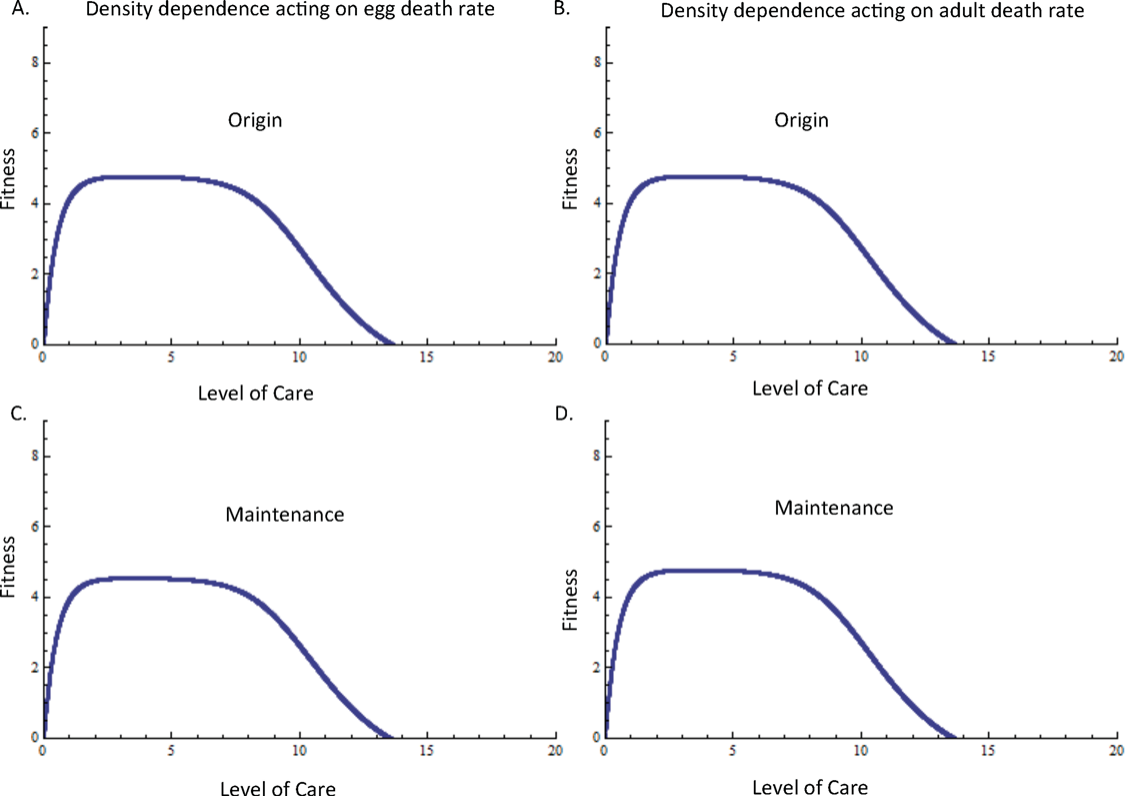
Fitness benefits of varying levels of parental care. Intermediate levels of care are favoured for the origin of parental care when (A) egg death rate and (B) adult death rate are density dependent. Intermediate levels of care are favoured for the maintenance of care when (A) egg death rate and (B) adult death rate are density dependent. *d*_*E* 0_ = *d*_*E m0*_ = 10, *m*_*E*_ = *m*_*E* m_ = 0.1, *r*_*0*_ = *r*_*m0*_ = 10,000, *d*_*A0*_ = *d*_*Am0*_ = 10, *σ*_*J0*_ = *σ*_*Jm0*_ = 0.01, *τ* = 0.1, *A*_*m*_ = 0–1.

For both the origin and maintenance of care scenarios, and for cases of either density dependent egg or adult mortality, intermediate levels of parental care result in the greatest fitness benefits (Fig 5A–5D). Indeed, relatively low and high levels of care will result in the smallest fitness benefits across scenarios (Fig 5). This pattern likely occurs because we assume non-linear benefits of care (i.e. the benefits of care in terms of decreased egg death rate depreciate in magnitude as care increases) and non-linear costs of care (i.e. the costs of care in terms of increased parental death rate and reduced reproduction begin to plateau as the level of care increases).

## Discussion

The evolution of parental care is influenced by basic life-history traits (Figs 1–4), but the specific life-history characteristics that lead to strongest fitness gains for care depend on 1) whether the origin or maintenance of care is being considered and 2) the life-history stage that density dependence acts on, in some cases (Figs 1–4; Table 2). In the early stages of the evolution of parental care, the strategy that exhibits care will be relatively rare in the population, and as a result, will experience competition primarily with resident individuals who do not exhibit care. However, as care spreads in a population and the number of individuals providing care increases, the mutant strategy will now experience competition with both non-caring resident individuals and itself (discussed in [7]). As such, it is somewhat unsurprising that the life-history conditions that favor care will depend on whether we are considering the origin or the maintenance of care.

**Table 2:** Overview of the life-history characteristics that favor the origin and maintenance of parental care when egg death rate or adult death rate are density dependent.

Life-History Trait	Density Dependent Scenario:	
	Egg Death is Density Dependent	Adult Death is Density dependent
Egg death rate (*d*_*E*_ and *d*_*Em*_)	**Origin of care:** Fitness benefits of care ↑ as egg death rate ↑. **Maintenance of care:** As the mutant strategy spreads in the population and mutant density increases, fitness benefits of care decrease but are insensitive to baseline egg death rate.	
Adult death rate (*d*_*A*_ and *d*_*Am*_)	**Origin and maintenance of care:** Fitness benefits of care ↑ when adult death rate is low.	**Origin of care:** No relationship between adult death rate and fitness benefits of care. **Maintenance of care:** As the mutant strategy spreads in the population and mutant density increases, fitness benefits of care are greatest when baseline adult death rate is low.
Juvenile survival rate (*σ*_*j*_ and *σ*_*jm*_)	**Origin of care:** No relationship between juvenile survival and fitness benefits of care. **Maintenance of care:** As the mutant strategy spreads in the population and mutant density increases, fitness benefits are greatest when juvenile survival is relatively high.	
Maturation rate of eggs (*m*_*E*_ and *m*_*Em*_)	**Origin and maintenance of care:** Fitness benefits of care are highest when egg maturation rate is highest.	**Origin and maintenance of care:** Fitness benefits of care are highest when egg maturation rate is lowest.
Juvenile stage development length (*τ* and *τ*_*m*_)	**Origin of care:** Fitness benefits of care are greatest when the juvenile stage is shortest. **Maintenance of care:** As the mutant strategy spreads in the population and mutant density increases, fitness benefits are greatest when the duration of the juvenile stage is relatively long.	

When considering the origin of parental care, the fitness benefits of care will be greatest when offspring need care the most—i.e. when egg death rate in the absence of care is relatively high—regardless of whether density dependence acts on the egg or adult stage (Fig 1A and 1B; Table 2). This finding is consistent with our previous theoretical work [17], which found that the origin of parental care will be most strongly favored when egg death rate in the absence of care, and hence offspring need, is high for the scenario in which competition affects adult reproduction (i.e. when adult reproduction is density dependent; [17]). This prediction is generally consistent with previous theoretical work [21] and the finding that parental care occurs relatively frequently in invertebrates when care is essential for survival [1]. More generally, these patterns suggest that parental care is most likely to originate in species in which offspring are highly unlikely to survive in the absence of care, such as insects, fish, or frogs that lay eggs in visible locations that have the potential to be predated upon or animals that lay eggs in relatively harsh physical environments (e.g. environments with low oxygen availability or high rates of disease).

In contrast, when we consider the maintenance of parental care, the fitness benefits of parental care are relatively insensitive to baseline egg survival (Fig 2A and 2B; Table 2). This suggests that once parental care begins to spread in a population, offspring need becomes relatively less important in maintaining parental care. Instead, baseline adult death rate begins to have a greater impact in promoting the maintenance of care as the care strategy becomes more common in the population. If one were to test these predictions across species, we might therefore expect to see 1) parental care originating when offspring survival in the absence of care is relatively low and offspring need is relatively high and 2) care being maintained regardless of whether offspring survival in the absence of care remains low or if offspring survival in the absence of care increases (e.g. due to other selection for increased offspring survival).

Importantly, our model did not assume that offspring need and parental care will co-evolve (i.e. baseline egg survival didn’t change through evolutionary time as care became more prevalent in the population). Some authors have hypothesized that care and offspring need will co-evolve, such that offspring will need (or solicit) more care once care is well-established in a system (reviewed in [8]; [45–47]). It is possible that such co-evolutionary dynamics will influence the conditions that favor parental care. While this is not considered in the current study, our model framework provides a way in which to explore the co-evolutionary dynamics of care with other life-history traits. Indeed, in systems in which parental care and offspring need co-evolve such that offspring become more dependent on care, we would expect parental care to continue to be maintained regardless of such co-evolution among care and survival.

We additionally found that the origin of parental care will be result in the greatest fitness benefits when the duration of the juvenile stage is relatively short regardless of whether density dependence acts on egg or adult mortality. This finding is consistent with our previous work on the origin of care in which we considered the scenario of density dependent adult reproduction [17]. However, in our current analyses, under the maintenance of care when mutants increase in density in the population, parental care is more likely to be maintained when the duration of the juvenile stage is relatively long (Table 2), as might be the case for some insects, fish, or mammals that experience delayed maturation. Likewise, when we considered the origin of care, the fitness benefits of egg-only care were unaffected by juvenile survival regardless of whether density dependence acts on egg or adult mortality (Table 2), and this pattern is consistent with the scenario in which density dependence acts on adult reproduction [17]. However, when we consider the maintenance of care, juvenile survival begins to affect the fitness benefits of parental care. Specifically, as mutant density increases, the maintenance of parental care will be most strongly favored when juvenile survival is relatively high for the scenarios of density dependent egg or adult mortality.

The specific patterns that we observed with regard to juvenile stage duration and juvenile survival are not entirely intuitive and are likely the result of the complex dynamics that occur in organisms that experience multiple life-history stages. Importantly, though, these patterns highlight that the conditions that favor the origin versus the maintenance of care can differ, and that once parental care increases in frequency, population-level dynamics can alter the conditions under which care will be promoted. Indeed, the finding that juvenile traits can influence the evolution of egg-only care is novel and potentially suggests that 1) complex life histories matter in relation to the evolution of parental care (i.e. an organism’s entire life history can influence the likelihood that care will originate and be maintained) and 2) relatively simplistic models that focus exclusively on the life-history stage in which care occurs might be missing some key drivers of parental care.

In some cases, the specific stage that density dependence acts on influences the conditions under which care is most likely to originate and be maintained. For example, when we consider the fitness benefit of parental care with respect to baseline adult death rate (i.e. adult death rate before any costs of care are accounted for), we find that the origin and maintenance of care will be most strongly favored when adult death rate is low when density dependence acts on the egg stage. Such a scenario would be expected to apply to animals that have relatively low competition for resources such as food or shelter or that exist at low densities. In contrast, when density dependence acts on adult mortality, baseline adult death rate has no effect on the fitness benefits associated with the origin of care (Table 2). This finding suggests that care can originate across a range of adult death rates in species that experience relatively intense adult competition for resources such as food or shelter. However, as care spreads, the fitness benefits associated with the maintenance of care will be greatest at lower baseline adult death rates when adult mortality is density dependent (Table 2), suggesting that care is most likely to be maintained in systems in which adults have relatively high survival when there is competition among adults for resources such as nests or food. These findings are in contrast to our previous work, which found that high baseline adult death rates favor the origin of parental care when adult reproduction is density dependent [17]. Combined, these findings suggest that parental care has the potential to originate and be maintained across a range of adult mortalities, and that details of stage-specific competition (e.g. stage-specific density dependence) can influence which specific life-history conditions will favor the origin and maintenance of parental care.

Similarly, stage-specific density dependence influences the maturation rates that are most likely to favor the origin and maintenance of care. When egg mortality is density dependent, care will be most strongly favored at high egg maturation rates (i.e. when individuals are rapidly developing and entering the juvenile stage); such a scenario might be expected to apply to fish and insects that lay eggs in close proximity if oxygen availability is limited or if disease has the potential to spread among offspring. The pattern of care resulting in the greatest fitness benefits when egg survival is density dependent and egg maturation rate is high is intuitive, as density dependent egg mortality will increase the number of offspring that die, and as such, it is beneficial for parents to provide care in order to increase survival of eggs during this relatively risky stage. Importantly, though, or modeling framework does not consider variation in egg density among nests in a population, and spatial variation in egg density might also affect the conditions under which care is most beneficial. In contrast to the scenario of density dependent egg mortality, when adult mortality is density dependent (Table 2) or when adult reproduction is density dependent [17], low egg maturation rates will favor the evolution of parental care. Such a scenario would be expected to occur in systems in which adults compete intensely for resources such as food or shelter. While the relationship between maturation rate and parental care has not been widely studied using a comparative approach, Winemiller and Rose [48] found that slow offspring growth (i.e. slower development) is associated with the occurrence of parental care in fishes. Thus, there is some evidence that offspring maturation rate can influence the evolution of parental care.

In general, the theoretical work presented herein suggests that the conditions that favor the origin and maintenance of care can differ. Most theoretical models of care focus only on either the origin or the maintenance of care, and our findings suggest that enhanced understanding of the evolution of parental care will require models that explicitly consider the evolutionary dynamics associated with both the origin and the maintenance of care (discussed further in [7]). Likewise, stage-specific density dependence can in some cases influence the life-history conditions that are most likely to favor the origin and/or the maintenance of care (summarized in Table 2). Broadly, these findings are consistent with the fact that parental care is found in animals that have incredibly diverse life histories [1, 8]. Indeed, our findings suggest that parental care can potentially originate and be maintained in a range of animal systems, and an obvious next step is to examine the relationship between basic life history-characteristics, stage-specific density dependence, and the origin and maintenance of care from a comparative perspective in various animal systems. Several studies have begun to use a comparative perspective to examine the evolution of different forms of care [2, 49–52]. For example, Gilbert and Manica [49] recently used a comparative approach to test hypotheses of the evolution of sex-specific patterns of parental care in insects. Such an approach could also be used in a range of systems to determine the life-history conditions (stage-specific rates of mortality and maturation) under which parental care is most likely to originate and be maintained in relation to differing ecological scenarios (e.g. density dependence acting on different life-history stages). In addition, it will also be important for future theoretical work to consider the full adaptive dynamics associated with the origin and maintenance of parental care. In the current paper, we used the relative growth rate of the mutant strategy as our measure of fitness; this measure provides insight into the fitness benefits of the mutant strategy when the resident strategy is in equilibrium, i.e. not growing. In the future, it will be important to consider the complete adaptive dynamics that will occur if and when the mutant invades the resident strategy, and the dynamics that occur when the resident attempts to re-invade the mutant strategy. Such a focus would provide a much more detailed look at when we would expect parental care to persist long term in a population and when care and no-care might be expected to co-exist.
